# Some Manifestations of Lithemia in the Young1Read at the annual meeting of the Medical Society of the County of Erie, January 9, 1900.

**Published:** 1900-06

**Authors:** Maud J. Frye

**Affiliations:** Buffalo, N. Y.


### SOME MANIFESTATIONS OF LITHEMIA IN THE YOUNG.[1]

By MAUD J. FRYE, M. D., Buffalo, N. Y.

THIS paper is based upon and, indeed, may be called a partial résumé of work done by B. K. Rochford, of Cincinnati, who is both pediatrist and physiologist, and whose interpretation of some of the disease manifestations in early life has the double merit of originality and helpfulness.

---

1. Read at the annual meeting of the Medical Society of the County of Erie, January 9, 1900.

Lithemia may be defined as an autointoxication, caused by the presence of an excess of alloxuric bodies in the body media. Some of these are uric acid, xanthin, hypoxanthin, heteroxanthin and paraxanthin. They are suboxidation products. Concerning the properties of members of this group, Dr. Rochford has this to say—the result of laboratory and clinical observation:

Uric acid and its compounds are not poisonous.

Paraxanthin and heteroxanthin are very poisonous leucomaines, not uncommonly associated in their elimination with uric acid. They are found in such small quantities in normal urine that their poisonous properties are lost in dilution.

During lithemic attacks, paraxanthin and heteroxanthin are excreted in enormous excess, the urine of these patients not containing such excess at any other time.

These bodies are excreted, but not formed by the kidneys. Their presence in the urine means that immediately prior to their excretion they were present in the blood. Paraxanthin and xanthin, separated from the urine of patients suffering from certain lithemic manifestations, are capable of producing in the mouse and guinea-pig a group of symptoms similar to those from which the patient suffered. No other poisons have been demonstrated in lithemic urine which are capable of producing the symptoms of lithemia.

The etiological factors in lithemia, which Dr. Rochford names are three—heredity, overfeeding and under exercise. Upon the first of these as a factor in early life he lays great stress: "Lithemia in infants and children always means that one or both parents suffer from some phase of the uric acid diathesis." He regards lithemia as a specific and not a degenerate inheritance.

A wide variety of symptoms are attributed to lithemia in the adult, and Dr. Rochford finds it the explanation of many ailments of early life. Three groups of cases, in which he considers the underlying cause to be lithemia, have seemed to me to be of especial interest. The first of these is named "Inanition Fever," by L. Emmet Holt. Description of it is as follows:

" Under this head are included cases seen during the first five days of life in which there is an elevation of temperature, apparently due to the fact that the infant gets very little, frequently nothing at all from the breast at which it is being suckled. . . . The symptoms presented by these infants were a hot, dry skin, marked restlessness, dry lips, and a disposition to suck vigorously anything within reach. With very high temperature there was considerable prostration and weakened pulse. With less severe cases there were only crying and restlessness. The rapidity with which the symptoms disappeared when the children were wet-nursed or properly fed was very striking."

Rochford interprets these not uncommon cases to be due to irritating uric acid deposits in the urinary passages of the infant. Crandall, writing under the title "Inanition Fever," in the *Archives of Pediatrics*, March, 1899, observed the symptom on which Rochford lays stress, that the urine in these cases is usually scanty, accompanied by copious discharge of urates at the height of the fever. Whether the explanation suggested by Dr. Rochford be the true one or not, it is interesting and reveals a hidden merit in the various teas with which the old-fashioned nurse loves to dose the new-born.

A second group of cases is styled by Rochford a lithemic gastric neurosis. This has been designated by others cyclic vomiting. Holt describes the disease as follows: "Characterised by periodical attacks of vomiting, recurring at intervals of weeks or months without any adequate exciting cause. The vomiting is severe and uncontrollable, and usually lasts from twelve hours to three days. It is attended with symptoms of general prostration, which may be alarming. The children who are subjects of it may show in the interval nearly all the signs of perfect health." Rochford gives the symptomatology thus: Lithemia may manifest itself in very young infants by attacks of gastric pains associated with rapid breathing, nausea, vomiting and fever. The gastric paroxysms may be so severe that all food is rejected for a period of from one to five days. The temperature may reach 104° or 105° F., but sometimes in the most severe cases the fever ranges between normal and 102°. In these attacks the infant may be prostrated to the last degree and there may be also much emaciation. Occasionally these lithemic paroxysms are ushered in by convulsions, which may in time come to be characteristic symptoms of these attacks. These gastric paroxysms are self-limited and are but little influenced by treatment. The stools following these attacks are very putrid and in young infants on a strictly milk diet are sometimes oily in character. Similar attacks usually styled gastralgia or enteralgia occur in the adult. In children these attacks tend to become of the true migrainous type in later life.

Dyspnea Rochford observed to be one of the most characteristic symptoms of paraxanthin poisoning. A form of this—very rapid breathing—is seen in combination with the gastric neurosis, not depending on fever and not explained by bronchial or pulmonary disease. He states that lithemic asthma occurs only in adults. Personally, I have seen a case of asthma occurring in a child of four of marked lithemic habit. This child in addition had adenoids, which were believed to be the chief cause of the asthma. He was not cured of the asthmatic attacks by their removal, though the operation, or the antilithemic treatment or both, somewhat reduced their frequency.

A further interesting observation made by this investigator is that the menstrual function is closely associated or coincident with the sudden appearance of a great excess of paraxanthin in the blood and urine of lithemic paitients—a possible explanation of some forms of dysmenorrhea. Thyroid feeding may result in the appearance of an excess of paraxanthin. Rochford suggests that a hyperactivity of the thyroid which its enlargement at the menstrual period makes probable may be the explanation of the increase of paraxanthin at that time.

It is hardly necessary to state that the urine of the lithemic attack is scanty, highly acid, of increased specific gravity, throwing down a deposit of urates on standing. In cases of migrainous gastric neurosis, Rochford has found albumin and hyaline casts, the urine becoming normal with the disappearance of the symptoms. The detection of paraxanthin is not practicable in ordinary work, the process being complicated and requiring about three weeks' time for its completion.

Rochford speaks of the tendency of one form of lithemia to pass into another as the child grows older. A little patient of mine in whom after some time I discovered the lithemic basis of his symptoms, began, when about nine months old, to suffer from periodical attacks of dyspnea, styled croup by his mother. I never saw him during one of these paroxysms and cannot describe their exact character. After a variety of remedies of greater or less uselessness had been tried by his physician and his mother, including a black silk string tied around his neck by the latter, the urine was examined, found of lithemic character, and antilithemic treatment instituted. The attacks of dyspnea practically disappeared, but the child now has at rather long interval a mild attack of lithemic gastric neurosis, vomiting everything taken for twelve or more hours. These attacks are accompanied by slight fever, and the usually limpid urine stains the napkin. At times some error in diet has been fancied to provoke the attack, but the severest one from which he has yet suffered was without explanation, aside from lithemia. The mother of this child gives a lithemic history.

If apology is needed for presenting so incomplete a paper, I offer as such the help which the recognition of the lithemic element in early life affords in managing many little patients.

224 ALLEN STREET.

